# Oxidative Stress as a Common Key Event in Developmental Neurotoxicity

**DOI:** 10.1155/2021/6685204

**Published:** 2021-07-19

**Authors:** Yuhei Nishimura, Yasunari Kanda, Hideko Sone, Hiroaki Aoyama

**Affiliations:** ^1^Department of Integrative Pharmacology, Mie University Graduate School of Medicine, Tsu, Mie 514-8507, Japan; ^2^Division of Pharmacology, National Institute of Health Sciences, Kawasaki, Kanagawa 210-9501, Japan; ^3^Yokohama University of Pharmacy, Yokohama, Kanagawa 245-0066, Japan; ^4^The Institute of Environmental Toxicology, Joso, Ibaraki 303-0043, Japan

## Abstract

The developing brain is extremely sensitive to many chemicals. Perinatal exposure to neurotoxicants has been implicated in several neurodevelopmental disorders, including autism spectrum disorder, attention-deficit hyperactive disorder, and schizophrenia. Studies of the molecular and cellular events related to developmental neurotoxicity have identified a number of “adverse outcome pathways,” many of which share oxidative stress as a key event. Oxidative stress occurs when the balance between the production of free oxygen radicals and the activity of the cellular antioxidant system is dysregulated. In this review, we describe some of the developmental neurotoxins that target the antioxidant system and the mechanisms by which they elicit stress, including oxidative phosphorylation in mitochondria and plasma membrane redox system in rodent models. We also discuss future directions for identifying adverse outcome pathways related to oxidative stress and developmental neurotoxicity, with the goal of improving our ability to quickly and accurately screen chemicals for their potential developmental neurotoxicity.

## 1. Introduction

The high prevalence of neurodevelopmental disorders such as attention-deficit hyperactive disorder (5.3%), autism spectrum disorder (1%), and schizophrenia (1%) is a source of increasing concern worldwide [1–3]. Many factors can be involved in the etiology of neurodevelopmental disorders, including genetic traits and *in utero* exposure to environmental contaminants and recreational drugs. Because the developing brain is generally more sensitive than the adult brain to toxicants, exposure to neurotoxic chemicals during development is considered to be a key factor in the prevalence of neurodevelopmental disorders [4–13]. Developmental neurotoxicity (DNT) can result in dysregulation of a range of processes in the brain, including neurogenesis, neuronal differentiation, synaptogenesis, and establishment of functional connectivity networks [7–9, 12]. Several molecular and cellular events leading to DNT, the so-called “adverse outcome pathways,” have been identified [14, 15]. These adverse outcome pathways cover a wide range of molecular initiating events, including inhibition of receptors and enzymes such as N-methyl-D-aspartate receptor and acetylcholinesterase and interruption of biosynthesis and bioavailability of thyroid hormone, which results in adverse outcomes such as impairment of cognitive functions, alteration of sensory functions, and impairment of motor functions [15]. Notably, many of these pathways share oxidative stress (OS) as a key common event associated with neurodevelopmental disorders [7, 8, 16, 17]. In this review, we describe how OS pathways are involved in DNT, describe representative examples of developmental neurotoxins (DTXs) and the mechanisms by which they affect cellular antioxidant and oxidant systems in rodent models, and discuss future directions to increase our understanding of adverse outcome pathways as they relate to DNT induced by OS.

## 2. The Role of Oxidative Stress in Developmental Neurotoxicity

OS occurs when reactive oxygen species (ROS) accumulate as a result of an imbalance between the production of ROS and the activity of the cellular antioxidant system [18, 19]. Superoxide anion (O_2_·^−^), one of the major cellular ROS, is generated as a byproduct of oxidative phosphorylation (OXPHOS) in mitochondria as well as through the activity of nicotinamide adenine dinucleotide phosphate (NADPH) oxidase (NOX) located at the plasma membrane [20, 21]. O_2_·^−^ is converted to hydrogen peroxide (H_2_O_2_) and then to oxygen (O_2_) and water (H_2_O) by enzymatic antioxidants, including superoxide dismutase (SOD), catalase (CAT), and glutathione peroxidase (GPX), and by nonenzymatic antioxidants such as glutathione (GSH). The correct spatiotemporal control of ROS production and activity is crucial to many physiological functions, including neuronal fate and development [22, 23]. However, excessive ROS levels result in damage to DNA, RNA, proteins, and lipids [18]. When this occurs in brain cells, the damage can adversely affect neuronal functions such as memory, learning, and cognition [14]. Various chemicals, both naturally occurring and man-made, can cause DNT through OS [7, 8]. In the following sections, we describe representative examples of DTXs that cause OS by targeting antioxidant enzymes (Table 1), mitochondria (Table 2), and/or NOX (Table 3).

### 2.1. DTXs Targeting Antioxidant Enzymes

O_2_·^−^ and H_2_O_2_ can create hydroxyl radicals that damage DNA, proteins, and lipids when present at aberrantly high levels [24]. O_2_·^−^ is produced by mitochondrial pathways and/or NOX and is converted to H_2_O_2_ by SOD. In turn, H_2_O_2_ is converted to O_2_ and H_2_O by CAT in peroxisomes or to H_2_O *via* the activity of GPX and oxidation of GSH to its disulfide form in the cytosol. Impairment of these antioxidant enzymes thus results in supraphysiological ROS levels that can cause DNT.

Perinatal or postnatal exposure of rodents to lead (Pb) has been shown to reduce the activities of antioxidant enzymes in different brain regions and causes behavioral impairments in the pups [25–28]. The mean blood Pb concentration of rat pups (5 *μ*g/dL) that showed decreased activity of antioxidant enzymes in the brain was equivalent to that of pregnant women who have a significantly high risk of preterm birth (≥5 *μ*g/dL, odds ratio 2) [29]. The divalent cation Pb^2+^ enters the cytosol and mitochondria *via* calcium ion (Ca^2+^) transporters and replaces other divalent cations such as Ca^2+^, zinc ion (Zn^2+^), selenium ion (Se^2+^), and iron ion (Fe^2+^), which are essential for the correct structure and activity of many antioxidant enzymes [24, 30–33]. Pb^2+^ also binds with high affinity to sulfhydryl (SH) groups and can inhibit key functional SH groups in antioxidant enzymes such as SOD, GPX, and CAT [30].

Exposure to methylmercury (MeHg) during early development also causes OS and impairs various neuronal functions, especially cerebellar control of movement, as shown in rodents [34]. MeHg binds with high affinity to both SH and selenol groups [34] and thus impairs the activities of antioxidant enzymes that require Se for their proper function, including GPX and thioredoxin reductase (TrxR) [24, 34, 35]. Perinatal exposure of mice to MeHg (5 ppm in drinking water) was shown to decrease TxrR activity in the cerebrum and cerebellum and GPX1 activity in the cerebrum of male, but not female, pups [36]. This sex difference in GSH and Trx metabolism may be related to the higher prevalence of neurodevelopmental disorders among males compared with females in humans [37, 38]. The median blood Hg concentration of rat pups at birth from dams exposed to MeHg (0.5 ppm in drinking water) during pregnancy was 3.5 and 4.0 mg/L for males and females, respectively [39]. The decline of differential reinforcement of high rates of behavior occurred sooner in the offspring of rat dams exposed to MeHg (0.5 ppm in drinking water) throughout gestation compared to the offspring of dams without MeHg exposure [40]. The total range of Hg concentrations in umbilical cord blood in a World Trade Center cohort, a Faroese birth cohort, and a cohort study of congenital Minamata disease was 0.1-63, 0.9-351, and 20-699 *μ*g/L, respectively [41–43]. Higher cord blood Hg concentration was associated with the decline of the developmental score at 3 and 4 years old [41].

Arsenic (As) is another element that inhibits antioxidant enzyme activity through modification of functional SH groups [44]. This has been demonstrated in rats, where perinatal exposure of dams to 2–100 mg/kg/day As from gestational day (GD) 6 to postnatal day (PND) 21 resulted in impaired SOD, CAT, GPX, and glutathione reductase activities in various brain regions of the pups [45, 46]. The 50% lethal dose of As in rats ranges from 15 to 293 mg/kg [47]. Children with water As levels > 50 *μ*g/L showed significantly low intellectual abilities than children with As levels < 5.5 *μ*g/L in Bangladesh [48]. Mouse pups from dams exposed to As (55 *μ*g/L in drinking water) during the perinatal period showed learning and memory impairment [49]. The effects of As exposure at these concentrations on the OS in the development of the central nervous system remain to be studied.

The effects of chemicals such as cypermethrin [50], opioids [51], and silver nanoparticles [52] at sublethal doses have been examined in rodents to elucidate the toxicological mechanisms related to OS. Although such concentrations may not be relevant to environmental exposure, the results of these studies suggest that attention should be paid to the consequences of OS after exposure to these chemicals at relatively low doses.

### 2.2. DTXs Targeting the Mitochondria

OXPHOS is the major cellular pathway of energy production and occurs *via* the coordinated transfer of electrons through five multisubunit complexes (I–V) located in the inner mitochondrial membrane [24]. Complexes I and II generate electrons through the conversion of nicotinamide adenine dinucleotide from its reduced form (NADH) to its oxidized form (NAD+) and of flavin adenine dinucleotide from its reduced form (FADH_2_) to its oxidized form (FAD+). Complex III passes these electrons through coenzyme Q to cytochrome c. Cytochrome c oxidase (complex IV), which contains two heme groups and two copper atoms, then transfers four electrons to one O_2_ molecule, resulting in the generation of two H_2_O molecules. Complexes I, III, and IV also pump protons from the mitochondrial matrix into the cristae. Complex V utilizes these protons to generate adenosine triphosphate (ATP) from adenosine diphosphate (ADP) and inorganic phosphate [24]. O_2_·^−^ is generated as a byproduct during the transport of electrons to O_2_. Thus, impairment of the functions of these complexes can thus result in abnormal O_2_·^−^ production, leading to OS.

Perinatal exposure of rats to As at concentrations that decreased the activities of antioxidant enzymes also resulted in inhibition of complex I, II, III, and IV activities in the frontal cortex, hippocampus, and corpus striatum of the pups [46]. This mechanism is known to be involved in the neurotoxicity of As [53–55]. Another possible mechanism of DNT is that exposure to As may decrease the expression of genes encoding the components of complexes I–V in the brain [56, 57].

Similar to the effects of As, perinatal exposure of rats to manganese (Mn) also inhibits complex II activity in the pup striatum, although, unlike the effects of As, complex I activity is increased by Mn [58]. In this study, the rap pups were intraperitoneally injected with Mn (5, 10, or 20 mg/kg) for five consecutive days [58]. In humans, oral ingestion of Mn (about 1.8 mg/kg) for 4 weeks caused muscle weaknesses and psychological alterations [59]. The inhibitory effect of Mn on complex II has been demonstrated to selectively occur in mitochondria in the brain, but not the heart or liver, of rats exposed to Mn [60]. However, the mechanism by which Mn affects complex II remains to be elucidated.

The structure of mitochondria is dynamically regulated by fission and fusion [61]. Dynamin-related protein 1 (Drp1) and mitofusin 1 and 2 (Mfn1 and Mfn2) play important roles in mitochondrial fission and fusion, respectively. Drp1 is recruited to the mitochondrial outer membrane, where it oligomerizes and assembles a scission machinery that enables organelle constriction and cleavage. Mfn1 and Mfn2 are located at the mitochondrial outer membrane and, together with optic atrophy 1 (Opa1) located in the inner mitochondrial membrane, mediate the stepwise events that result in mitochondrial fusion. Mitochondrial fission can be triggered by various stimuli, including 1-methyl-4-phenylpyridinium and isoniazid, which affect the mitochondrial membrane potential [62, 63]. Aberrant stimulation of mitochondrial fission and fusion creates a deleterious cycle resulting in excessive ROS production [64].

A number of exogenous chemicals have been shown to modulate mitochondrial dynamics in the developing rodent brain. Postnatal exposure of rodent pups to the inhalation anesthetic sevoflurane increases and decreases the expression of Drp1 and Mfn2, respectively, in the hippocampus [65] and additionally increases the expression of cyclophilin D, a factor that modulates the mitochondrial permeability transition pore [66]. The concentration of sevoflurane used in these rodent studies (3%), which did not induce significant disturbances in ventilation, blood oxygenation, or cerebrospinal fluid content in mice [67], is similar to the minimum alveolar concentration of sevoflurane used for anesthesia in children (2.0-3.3%) [68]. Postnatal exposure of rat pups to general anesthesia, composed of midazolam, isoflurane, and nitrous oxide, increases the expression and oligomerization of Drp1 at the mitochondria to promote fission in the subicular region of the pup brain [69] and additionally increases mitochondrial membrane permeability [70]. The concentration of isoflurane used in the rat study (0.75%) is similar to those that caused isoflurane-induced neuroapoptosis in the neonatal rhesus macaque brain (0.7-1.5%) [71]. These results suggest that changes in mitochondrial membrane permeability may be involved in the mechanism for the anesthesia-induced enhancement of mitochondrial fission in the developing brain. In addition to the *in vivo* studies, Mfn has been reported to mediate neural differentiation from human induced pluripotent stem cells, which are commonly used for DNT evaluation *in vitro* [72, 73]. Several DTXs, including tributyltin and 5-fluorouracil, have been reported to induce degradation of Mfn protein and subsequently inhibit neural differentiation [72, 74]. Thus, it would be useful to include examination of mitochondrial dynamics in the evaluation of DTXs *in vivo* and *in vitro*.

### 2.3. DTXs Targeting NOX

The NOX family of enzymes is located on the plasma membrane and is an important source of ROS, especially O_2_·^−^ [75, 76]. The human NOX protein family consists of seven homologs: NOX1, NOX2, NOX3, NOX4, NOX5, DUOX1, and DUOX2. NOX1, NOX2, and NOX3 interact with p22phox, a transmembrane protein, and act as a scaffold and binding platform for the cytosolic activators Noxo1, Noxa1, p47phox, p67phox, and Rac [75]. DUOX1 and DUOX2 require binding to Duoxa1 and Duoxa2, respectively, to exert their activities.

Postnatal exposure of mouse pups to sevoflurane increases the expression of NOX2 [77] and p22phox [78], resulting in OS and apoptosis in the brains. Notably, these experiments were performed with sevoflurane concentrations (3%) comparable to those used for anesthesia in children (2.0-3.3%) [68]. In the mouse experiments, the effects were ameliorated by coadministration of the NOX inhibitor apocynin [78] or curcumin [77], suggesting that sevoflurane-induced OS in the developing brain is caused by activation of NOX, at least in part [79]. Further work will be needed to fully understand the mechanisms by which sevoflurane activates NOX.

Exposure of the fetus to ethanol has a profound effect on the developing brain; indeed, OS resulting from prenatal exposure to ethanol is a key pathogenic factor in fetal alcohol syndrome [80]. Prenatal exposure of mice to ethanol increases brain expression of various NOX subunits, including p22phox, Noxa1, Noxo1, p67phox, and Rac1 [81, 82]. In these studies, the pregnant mice were intraperitoneally injected with ethanol at GD8 (12 g/kg) [81] or GD9 (2.9 g/kg) [82]. In primates, the majority of studies on fetal alcohol syndrome utilize oral intubation of ethanol (0.3-5.0 g/kg) once-weekly [83]. *In vitro* studies with the human neuroblastoma cell line SH-SY5Y showed that ethanol increases ROS production by inducing p47phox [84]. Of note, dominant negative inhibition of Cdc42 suppressed the induction of p47phox in SH-SY5Y cells, suggesting a potential role for Cdc42 in the effects of ethanol on NOX activity in the brain [84]. Nevertheless, the exact mechanisms by which ethanol exposure increases OS in the developing brain remain to be fully elucidated.

## 3. Future Directions

Supraphysiological intracellular levels of ROS can be generated through a number of mechanisms, including alterations in the balance between antioxidant and oxidant systems, perturbation of mitochondrial dynamics, and activation of NOX activity at the plasma membrane. As described here, excessive ROS levels negatively affect crucial neuronal functions in the developing brain that impact both neurodevelopmental and neurobehavioral pathways.

In this review, we mainly focused on studies that employed rodent models to examine DNT related to OS. In extrapolating the results to humans, however, it should be noted that several relevant species differences exist, including the structures and functions of the placenta [85, 86] and brain [87, 88]. For example, human placenta is hemomonochorial and includes a single syncytiotrophoblast zone, whereas mouse placenta is hemotrichorial and consists of a trophoblast giant cell layer, spongiotrophoblast layer, and labyrinthine layer [85]. Additionally, human placenta, but not mouse placenta, contains an aromatase enabling the synthesis of estrogen [86]. The major growth spurt of the brain occurs at different stages in humans and rats, namely, at the prenatal and postnatal stages, respectively [88]. Various human testing platforms, such as placenta-on-a-chip and the *ex vivo* placenta perfusion model, as well as brain microphysiological systems, have been developed to more precisely assess DNT of chemicals in humans [15, 85, 89, 90]. The difference of DNT between prenatal and postnatal exposure in human should also be carefully examined [91].

The upstream and downstream events resulting in and from abnormal ROS levels can be investigated *in vitro* using neuronal cells that differentiated from embryonic stem cells or induced pluripotent stem cells [72, 74, 92, 93]. Studies using human neural stem cells, for example, have revealed that rotenone, an inhibitor of mitochondrial complex I, activates the Nrf2 pathway in response to OS and that activation of this pathway could be used as a readout to assess neurotoxicity [94]. These experimental systems enable testing of the major neurodevelopmental endpoints included in a DNT testing battery [73].

Alternative *in vivo* testing methods that employ fish, which are commonly used to examine morphological and behavioral effects, can be employed to assess DNT mediated by OS [95–98]. In one example, developmental exposure of zebrafish to endosulfan, a fat-soluble organochlorine pesticide, caused morphological defects and abnormal behavior that was suppressed by coexposure to vitamin E, a fat-soluble antioxidant, suggesting that OS was a key event in endosulfan neurotoxicity [99]. Several zebrafish behaviors have also been used as alternative phenotypes of neurodevelopmental disorders in humans [95, 100].

Neuroinflammation is closely related to OS and associated with neurodevelopmental disorders [101–104]. Supraphysiological intracellular levels of ROS induce the production of proinflammatory cytokines from astrocytes and microglia [101, 104]. These cytokines activate NOX of astrocytes and microglia, resulting in a vicious cycle of OS and neuroinflammation [103, 105]. The neuroinflammation may lead to neuronal apoptosis and alterations of homeostatic levels of neurotransmitters [102, 104]. Neuroinflammation can be assessed in rodents [106], zebrafish [107], and pluripotent stem cells [108].

Various omics technologies are powerful strategies to identify adverse outcome pathways [109–114]. For example, integration of cell-based high-throughput screening, cell lysate microarray immunostaining, and transcriptome analysis successfully identified OS as a key common adverse outcome pathway in nanomaterial-induced fibrosis and cancer [115]. *In silico* analysis also provides useful information about the pharmacokinetics of chemicals that cause OS [116] as well as their ability to induce ROS [117, 118] and their quantitative structure-activity relationships [119]. Integration of *in silico*, *in vitro*, and/or *in vivo* studies will also facilitate prediction of the toxicities of OS-related chemicals [120–123].

Integration of these approaches will not only enhance our understanding of OS-related DNT and the relationship between dysregulated OS and the pathogenesis of neurodevelopmental disorders but also expand our ability to quickly and accurately screen chemicals for their potential DNT properties.

## Figures and Tables

**Table 1:** Developmental neurotoxins targeting antioxidant enzymes.

Chemical	Exposure, species	Toxicities on antioxidant enzymes in the developing brain	Other findings in pups	References
Pb	Pregnant rats were allowed access to 0.1% PbAc in drinking water ad libitum from GD1 to PND21. Blood and brain Pb concentrations in the pups at PND28 were 5 *μ*g/dL and 7 *μ*g/g dry mass, respectively	Both SOD2 and GPx activities were decreased in FC, HC, and CB on PND28. Both SOD1 and CAT activities were decreased in HC on PND28	The concentrations of Se, Zn, Cu, and Mn were decreased in FC, HC, and CB on PND28	[25]
Pb	Pregnant rats were given access to drinking water containing 0.2% PbAc from GD5 to PND21. Brain Pb concentrations at PND21 were ~20 *μ*g/g	Both SOD and GPx activities were decreased in CB shortly after exposure	Locomotor activities were impaired on PD31-33. Purkinje cell densities were decreased in PD33. Coadministration of melatonin alleviated the DNT of Pb	[26]
Pb	Pregnant rats were given access to drinking water containing 0.2% PbAc from GD6 to PND21	The activities of SOD1, CAT, GPX, and XO were decreased in both HC and CB on PND21, 28, 35, and 60	Calcium supplementation ameliorated the DNT of Pb	[27]
Pb	Male rats, aged 4-5 weeks, were injected IP with PbAc at 10–60 mg/kg once daily for 5 days	SOD activity was decreased in both FC and HC shortly after exposure	Bax expression and neuronal apoptosis were increased in FC and HC shortly after exposure. Coadministration of t-BHQ (activator of Nrf2) suppressed the DNT of Pb	[28]
MeHg	Pregnant mice were given access to drinking water containing 5 ppm MeHg (~400 *μ*g/kg/day) from postmating to PND21	TrxR activity was decreased in both the cerebrum and CB in male on PND21. GPx activity was decreased in the cerebrum in male on PND21	TrxR and GPx activities in the cytoplasmic extract of CB were increased in female on PND21	[36]
As	Pregnant rats were given access to drinking water containing As 100 ppm from GD6 to PND21	The activities of SOD1, SOD2, CAT, GPX, and GR were decreased in CC, HC, and CB on PND21, PND28, and 3 months old	Lipid peroxidation and the expression of caspase-3/9 mRNA were increased in CC, HC, and CB on PND21, PND28, and 3 months old	[45]
As	Pregnant rats were injected IP with 2–4 mg/kg As once daily from GD6 to PND21	CAT activity was decreased in FC, HC, and CS on PND22 and 45. SOD activity was decreased in FC, HC, and CS on PND22	Expression of Bax and caspase-3 proteins was increased in FC, HC, and CS on PND22 and 45	[46]

GD: gestational day; PND: postnatal day; TrxR: thioredoxin reductase; GPX: glutathione peroxidase; SOD: superoxide dismutase; GR: glutathione reductase; FC: frontal cortex; CC: cerebral cortex; HC: hippocampus; CB: cerebellum; CS: corpus striatum; IP: intraperitoneal.

**Table 2:** Developmental neurotoxins targeting mitochondria.

Chemical	Exposure, species	Toxicities on mitochondria in the developing brain	Other findings in pups	References
As	Pregnant rats were injected IP with 2–4 mg/kg As once daily from GD6 to PND21	Complex II and III activities were decreased in FC, HC, and CS on PND22 and 45. Complex I and IV activities were decreased in FC, HC, and CS on PND22	ROS and MMP were increased and decreased, respectively, in FC, HC, and CS on PND22 and 45	[46]
Mn	Pups were injected IP with MnCl_2_ (5–20 mg/kg) once daily from PND8 to PND12	Complex I and II activities were increased and decreased, respectively, in the striatum of the pups on PND14	ROS and caspase activity were increased in the striatum of the pups on PND14. Abnormalities in motor coordination were observed at 3-5 weeks of age	[58]
Sevoflurane	Rats at PND7 were anesthetized with 3% sevoflurane in 40% oxygen for 4 h	The protein expression of Drp1 and Mfn2 was increased and decreased, respectively, in HC shortly after exposure	Cleaved caspase-3, cytochrome c, and apoptosis were increased in HC shortly after exposure. Abnormalities in spatial learning and memory were observed at PND30	[65]
General anesthesia (midazolam, isoflurane, and nitrous oxide)	Rats at PND7 were injected IP with midazolam (9 mg/kg) and then exposed for 6 h to nitrous oxide (75%), isoflurane (0.75%), and oxygen (approximately 24%)	Expression and oligomerization of Drp1 protein in mitochondria were increased in subicular and thalamic regions shortly after exposure	ROS and fission of mitochondria in the subicular region were increased shortly after exposure	[69]

GD: gestational day; PND: postnatal day; FC: frontal cortex; HC: hippocampus; CS: corpus striatum; MMP: mitochondrial membrane potential; IP: intraperitoneal.

**Table 3:** Developmental neurotoxins targeting NADPH oxidase.

Chemical	Exposure, species	Toxicities on NOX in the developing brain	Other findings in pups	References
Sevoflurane	Mouse pups at PND6 were anesthetized with 3% sevoflurane in 40% oxygen for 6 h	p22phox protein expression was increased in the brain shortly after exposure	ROS, cytochrome c, and cleaved caspase-3 were increased in the brain shortly after exposure. Abnormal freezing behavior was observed at 11-13 weeks of age. These toxicities were suppressed by cotreatment with the NOX inhibitor	[78]
Sevoflurane	Mouse pups were anesthetized with 3% sevoflurane in 40% oxygen for 2 h daily from PND6 to PND8	NOX2 protein expression was increased in FC and HC shortly after exposure	Apoptosis was increased in the brain shortly after exposure. Abnormal freezing behavior and the impairments of spatial learning and memory were observed at 9-11 weeks of age	[77]
Ethanol	Pregnant mice at GD8 were injected IP with ethanol (12 g/kg)	The mRNA expressions of Duox2, Noxa1, and Noxo1 were increased in the brains on GD18	The mRNA expressions of Noxa1 and p67phox were increased in the placenta and liver, respectively, on GD18	[81]
Ethanol	Pregnant mice at GD9 were injected IP with ethanol (2.9 g/kg)	The mRNA expressions of Duox1, Noxa1, Noxo1, p22phox, p67phox, and Rac1 were increased in the brains shortly after exposure	NOX activity, ROS generation, oxidative DNA damage, and apoptosis were increased in the brains shortly after exposure. These toxicities were suppressed by cotreatment with the NOX inhibitor	[82]

GD: gestational day; PND: postnatal day; FC: frontal cortex; HC: hippocampus; IP: intraperitoneal.
